# Supplementary material for: The Limited Palette for Photonic Block‐Copolymer Materials: A Historical Problem or a Practical Limitation?
Source: Angew Chem Int Ed Engl. 2022 Apr 21;61(22):e202117275. doi: 10.1002/anie.202117275 (PMC9325480; doi:10.1002/anie.202117275)
Supplement: Supplementary file 1 — Supporting Information [file ANIE-61-0-s001.pdf]

Angewandte Chemie
Eine Zeitschrift der Gesellschaft Deutscher Chemiker

Supporting Information

The Limited Palette for Photonic Block-Copolymer Materials: A Historical Problem or a Practical Limitation?

Z. Wang, C. L. C. Chan, R. M. Parker, S. Vignolini**

Section S1. The optical behavior of photonic multilayer films.

For photonic multilayer films, the key parameters dictating the characteristic wavelength of the photonic multilayer film (λ_{max}) are the domain spacing (d) and the average refractive index (n_{av}). Under normal incidence, the characteristic wavelength can be estimated from the Bragg equation (**Equation 1**). However, for lamellae with asymmetric domains or when viewed at other angles of incidence, the Bragg-Snell equation, which accounts for both the angular dependence of the wavelength of the reflected light and refraction at the air-multilayer interface, should be employed instead (**Equation S1**):[1]

$$m\lambda_{max} = 2(d_1 + d_2)\sqrt{\frac{(n_1 d_1 + n_2 d_2)^2}{(d_1 + d_2)^2} - \sin^2 \theta} \tag{S1}$$

where n_i and d_i are the refractive index and thickness of a domain of block i, θ is the angle of incident light onto the multilayer film and m is the order of reflection (typically only the first order is considered, i.e., $m = 1$).

Section S2. Abbreviations and full names of (i) linear block copolymers; (ii) backbones, side chains, or polymers in brush block copolymers; (iii) tuning methods and (iv) common solvents for structurally colored films.

Table S1: Linear block copolymers and their corresponding abbreviation.

**Abbreviation**	**Linear block copolymer name**
PS-*b*-P2VP	polystyrene-*b*-poly(2-vinylpyridine)
PS-*b*-PI	polystyrene-*b*-polyisoprene
PS-*b*-PE/P	polystyrene-*b*-poly(ethylene propylene)
PS-*b*-P4VP	polystyrene-*b*-poly(4-vinylpyridine)
PS-*b*-P*t*BMA	polystyrene-*b*-poly(*tert*-butyl methacrylate)
PS-*b*-PMAA	polystyrene-*b*-poly(methacrylic acid)
PS-*b*-PMMA	polystyrene-*b*-poly(methyl methacrylate)
P(E-*co*-P)-*b*-P(E-*co*-N)	poly(ethylene-*co*-propylene)-*b*-poly(ethylene-*co*-norbornene)
P(MCP-*co*-VTM)-*b*-P(E-*co*-N)	poly(methylenecyclopentane-*co*-3-vinyltetramethylene)-*b*-poly(ethylene-*co*-norbornene)
LLDPE-*b*-ULDPE	linear low-density polyethylene-*b*-ultra low density polyethylene
PS-*b*-PSM	polystyrene-*b*-poly(solketal methacrylate)
PTEPM-*b*-PSMA	poly(3-(triethoxysilyl)propyl methacrylate)-*b*-poly(stearyl methacrylate)
PI-*b*-P4MS	polyisoprene-*b*-poly(4-methylstyrene)
PS-*b*-PHEMA	polystyrene-*b*-poly(2-hydroxyethyl methacrylate)
PBD-*b*-PEO	poly(1,2-butadiene)-*b*-poly(ethylene oxide)

Table S2: Name and corresponding abbreviation of backbones, side chains, or polymers used to prepare brush block copolymers.

**Abbreviation**	**Full name**
P(PS)-*b*-P(PDLLA)	polystyrene-*b*-poly(DL-lactide)
P(PLLA)-*b*-P(PVP)	poly(L-lactide)-*b*-poly(N-vinylpyrrolidone)
I (or NBI)	norbornene imide
N (or NB)	norbornene
PS	polystyrene
P*t*BOS	poly(4-*tert*-butoxystyrene)
PHS	polyhydroxystyrene
PMMA	poly(methyl methacrylate)
P*t*BMA	poly(*tert*-butyl methacrylate)
P*t*BA	poly(*tert*-butyl acrylate)
PEO	poly(ethylene oxide)
P*n*BA	poly(*n*-butyl acrylate)
PHI	poly(hexyl isocyanate)
P4PBI	poly(4-phenyl butyl isocyanate)
AW	alkyl wedge
B*n*W	benzyl wedge
DDW	dodecyl wedge
FB*n*W	fluorobenzyl wedge
BzM	benzyl monomer
AM	alkyl monomer
DMA	dodecyl methacrylamide
BW	benzyl wedge with second-generation dendrons
POSS	polyhedral oligomeric silsesquioxane
PBzMA	poly(benzyl methacrylate)
PDMS	polydimethylsiloxane
FeCp	ferrocene group
TPE	tetraphenylethene

Table S3: Tuning methods used to control the structural color of photonic BCP materials, and their abbreviation.

**Abbreviation**	**Swelling method**	**Notes**
BCPB	Block copolymer blend	A blend of two or more block copolymers
BTF	Biotin functionalization	
CBBDP	Changing backbone DP	
CD	Chemical doping	Blending with functional molecules
CGD	Changing grafting density	
CHGST	Changing homogenization speed and time	
CHGST-Dry	Changing homogenization speed and time, and after drying	
CI	Counter ions	Quaternized or ionized using salts
CL	Crosslinking	
CPS	Changing preparation solvent	
CSC	Changing side chain	
CSET	Changing solvent evaporation time	
CVF-PEO	Changing volume fraction of PEO	
EF	Electrical field	
HPB	Homopolymer blend	Blends with homopolymers or with cellulose nanofibers (indicated with * in **Table S5**)
MF	Mechanical force or strain	
MID	Metal ion doping	
ND	Nanoparticle doping	
RMB	Reactive monomer	
RR-H_2O_2	Redox reaction with hydrogen peroxide	
RR-Salt	Redox reaction with salt	
SH	Shearing	
SMC	Supramolecular chemistry	
SS	Solvent swelling	
TC	Temperature change	
UVLE	Ultraviolet light exposure	

Table S4: Common solvents used to cast BCP films, and their abbreviation.

**Abbreviation**	**Solvent**
TCE	1,1,2-trichoroethane
PGMEA	propylene glycol monomethyl ether acetate
THF	tetrahydrofuran
DVB	divinylbenzene
THOH	THFFA-HDA-ODA-HDDA, a mixture of acrylate monomers composed of tetrahydrofurfuryl acrylate, hexadecyl/octadecyl acrylate, and 1,6-hexanediol diacrylate
DDC	DOP-DMP-C14, a mixture composed of di-*n*-octyl phthalate, dimethyl phthalate, and *n*-tetradecane
DCM	dichloromethane

Section S3. Statistics of linear and brush block copolymers employed for photonic multilayer films or photonic particles (pigments).

Clarification of the fields used to classify block copolymers used for photonic materials:

[a] **Name:** The linear block copolymers are named according to the convention: (first block)-*b*-(second block). The abbreviations are defined in **Table S1**. The brush block copolymers were named according to the rule: P(backbone-side chain 1)-*b*-P(backbone-side chain 2). The abbreviations are defined in **Table S2**. Additionally, the refractive indices of the blocks are given in parenthesis in the form: (Refractive index of block#1-Refractive index of block#2). All LBCPs are synthesized by anionic polymerization unless explicitly stated. Similarly, all BBCPs are prepared via ROMP of MMs unless explicitly stated otherwise.

[b] **M_n (kDa):** The number average molecular weight (M_n) was taken directly from the articles where available. If unavailable, they were calculated from other parameters presented, for example, weight average molecular weight (M_w) and polydispersity index (PDI). "N/A" indicates that the M_n could not be obtained. For brush block copolymers, this also includes molecular weights (M_n) of the side chains (SC_1 and SC_2).

[c] **PDI:** The polydispersity index (PDI) was taken directly from the articles where available. If unavailable, they were calculated from other parameters provided, for example, weight average molecular weight (M_w) and number average molecular weight (M_n). "N/A" indicates that the PDI could not be obtained. The PDI was rounded to two decimal places.

[d] **DP_1, DP_2, and DP_1/DP_2:** The degree of polymerization (DP) is provided for each block, (i.e., DP_1 and DP_2 for block#1 and block#2, respectively) and were either taken from the articles or, if not directly available, estimated from other parameters presented, for example, the ratio of the molecular weight of each block and that of the respective monomer. "N/A" indicates that the DP could not be obtained. The ratio DP_1/DP_2 were calculated to one decimal place.

[e] **Formation method:** The method by which the lamellar structure was produced from the BCP.

[f] **Tuning method:** The method used to tune the characteristic wavelength, typically through altering the native domain spacing. The abbreviations are defined in **Table S3**. A dash is used to show that several treatments were applied. "N/A" indicates that the

tuning methods were not reported in the article.

[g] **Solvent:** The preparation solvent from which the photonic BCP film was drop-cast. The abbreviations are defined in **Table S4**. "N/A" represents that no solvents have been used (i.e., the film was cast from a BCP melt) or the used solvents were not stated in the article.

[h] **d (nm) or 2ξ (nm):** The domain spacing d of the formed lamellar structure, defined as the total thickness of a layer comprised of one domain of block#1 and one domain of block#2 (i.e., $d = d_1 + d_2$, as defined in **Figure 1a** of this article). For photonic porous particles (as in **Table S9**), the center-to-center correlation distance, 2ξ, is stated as the photonic dimension. "N/A" indicates that no specific domain spacing value was reported. "Native" domain spacing indicates that the value was measured from a dry film in the absence of any further treatment, such as solvent swelling. As different methods were used to measure and evaluate domain spacings, the value presented was chosen according to the following order of priority if more than one measurement technique was reported: XS (X-ray scattering), TEM (transmission electron microscopy), SEM (scanning electron microscopy), AFM (atomic force microscopy), NS (neutron scattering), other techniques. It should be noted that this order was chosen to simplify the information presented and does not necessarily comment on the relative accuracy of these techniques. In the case where more than one value was available using the same measurement method, both measurements will be exhibited (separated by a slash, /). If values were not given explicitly, they were extracted from the figures in the source article. The domain spacing is given to the nearest integer.

[i] **$\lambda_{\max}$ (nm):** The characteristic wavelength $\lambda_{\max}$ is the wavelength at which the maximum intensity of light is reflected by the photonic structure. Conversely, when reflection spectra were not provided, this could also be determined from the position of the dip in the transmission spectrum. "N/A" indicates that no such wavelength was reported. "UV" indicates that the film reflected within the ultraviolet region and therefore appeared colorless. "Native" represents that the value of the maximum characteristic wavelength was measured from a dry film without any further treatment, such as solvent swelling. As different methods were used to measure and evaluate the wavelength, the value presented was chosen according to the following order of priority if more than one measurement technique was available: reflection spectroscopy, transmission or absorption spectroscopy. It should be noted that this order was chosen to simplify the information presented and does not necessarily comment on the relative

accuracy of the techniques. In the case where more than one value was available using the same measurement method, both measurements will be exhibited (separated by a slash, /). If values were not given explicitly, they were extracted from figures in the source article. The characteristic wavelength is given to the nearest integer.

Table S5: Linear block copolymers for photonic multilayer films.

BCP properties							Self-assembly conditions			Optical characteristics	
Name[a]	Articles (#)	M_n[b] (kDa)	PDI[c]	DP_1[d]	DP_2[d]	$\frac{DP_1}{DP_2}$[d]	Formation method[e]	Tuning method[f]	Solvent[g]	*d*[h] (nm)	λ_{max}[i] (nm)
PS-*b*-P2VP	1[2]	443	N/A	2381	1855	1.3	Annealing	SS	TCE; PGMEA; Toluene; 1,4-Dioxane; THF	N/A	UV (native)-646
	6[3]	380	1.10	1824	1807	1.0		CL-CI-SS		100 (native)-640	UV (native)-1627
								CI-SS		N/A	310-1821
								SS		330	928
								ND		110-295	381-741
								SS-EF		≥ 90	UV-633
								CI-SS-EF		N/A	486-664
	1[4]	266	N/A	1277	1265	1.0		CL-CI-SS		N/A	421-747
								CI-SS			510-586
	1[5]	265	1.07	1277	1255	1.0		N/A		N/A	N/A
	2[6]	264	N/A	1277	1246	1.0		CL-CI		N/A	410
								CL-CI-SS			525-530
								SS			600
								CI-SS			590
	1[7]	260	1.10	1248	1236	1.0		CI-SS		N/A	312-1064
	1[8]	230	1.04	1015	1182	0.9		CL-CI-SS		93 (native)-210	UV-762
	8[9]	199	1.12	979	923	1.1		N/A		48/56 (native)	UV (native)
								SS-EF		N/A	475-675
								SS-MF			UV-760
								CI-SS			329-774
								CL-CI-SS			447-531
								SS		N/A	296-700
	2[9f, 10]	153	1.18	807	656	1.2		CI-SS		N/A	490-600
								SS			520-570
	1[11]	158	1.14	739	770	1.0		SS		57 (native)-202	UV (native)-618
	2[9e, 12]	142	N/A	720	632	1.1		SS		N/A	500-540
	1[13]	121	1.07	677	480	1.4		N/A		55 (native)	UV (native)
								SS		114-210	341-624
	1[14]	130	1.03	611	632	1.0		N/A		60 (native)	UV

BCP properties							Self-assembly conditions			Optical characteristics	
Name[a]	Articles (#)	M_n[b] (kDa)	PDI[c]	DP_1[d]	DP_2[d]	$\frac{DP_1}{DP_2}$[d]	Formation method[e]	Tuning method[f]	Solvent[g]	*d*[h] (nm)	λ_{max}[i] (nm)
								CL-CI-SS		N/A	461-734
								CL-CI-SS-MF		≥ 175	500-620
	1[15]	129	1.05	582	651	0.9		CL-CI-TC-SS		N/A	430-612
								CL-CI-SS		N/A	435-679
	1[16]	125	1.05	566	628	0.9		N/A		70 (native)	UV
								CI-TC-SS		N/A	728-990
								CL-CI-TC-SS			303-822
	1[8]	125	N/A	N/A	N/A	N/A		CL-CI-SS		≤ 263	≤ 600
	1[17]	126	1.05	568	635	0.9		CL-CI-SS		N/A	350
								CL-CI-SS-UVLE		≥ 151	460-1062
	21[3b, 3c, 9e-h, 10, 12, 18]	114	1.05-1.08	547	542	1.0		SS		N/A	380-719
								CI-SS-EF			419-610
								N/A		48/50/64 (native)	UV (native)
								CI-SS		N/A	363-922
								CI			366-373
								CI-SS-UVLE			372-836
								TC-SS		N/A	466-725
								BCPB-SS			420-680
								CI-BTF-SS			498-583
								N/A			280 (native)
								MID			292-1092
								CI		47	N/A
								CI-RMB-UVLE		50-53	N/A
								CI-RMB-SS-UVLE		N/A	416-525
								CL-CI-SS		N/A	403-587
	1[11]	105	1.05	528	476	1.1		SS		43 (native)-153	UV-469
	1[19]	111	N/A	528	533	1.0		N/A		60 (native)	UV
								SS		N/A	477-647
	9[5, 20]	109	1.07-1.14	499	542	0.9		CI-SS-UVLE		N/A	508-594
								CI-SS			393-788
								CI-RMB-SS			405-662

BCP properties							Self-assembly conditions			Optical characteristics	
Name[a]	Articles (#)	M_n[b] (kDa)	PDI[c]	DP_1[d]	DP_2[d]	$\frac{DP_1}{DP_2}$[d]	Formation method[e]	Tuning method[f]	Solvent[g]	*d*[h] (nm)	λ_{max}[i] (nm)
								CI-RMB-SS-UVLE			436-662
								CI-SS-EF			538-604
								CI -SS-EF-CD			394
	1[21]	105	1.05	480	523	0.9		N/A		60 (native)	N/A
								CL-CI-SS		N/A	330-810
	1[8]	90	N/A	N/A	N/A	N/A		CL-CI-SS		≤ 177	≤ 400
	1[22]	81	1.04	373	401	0.9		N/A		30 (native)	UV
								CI-SS		150-212	307-580
								CI-SS-EF		N/A	500-604
	1[8]	80	1.05	361	403	0.9		CL-CI-SS		45 (native)-91	UV-388
	1[11]	78	1.07	365	380	1.0		N/A		37 (native)	UV (native)
								SS		137	379
								BCPB-SS		N/A	442-558
	1[23]	N/A	N/A	N/A	N/A	N/A		SS		N/A	372-764
PS-*b*-PI	1[24]	1410	1.04	7585	9102	0.8	Shearing	CL-SS	DDC; THOH; Toluene; Cumene; o-Xylene; DVB	205	601
	1[25]	1005	1.02	4753	7487	0.6	Solvent-evaporation	N/A		164 (native)	N/A
							Dissolving in solution	SS		173-216	521-655
							Shearing	SS		N/A	455-666
	1[26]	1040	1.02	4609	8221	0.6	Dissolving in solution	SS		168-192	N/A
	3[27]	840	1.08	4609	5285	0.9	Solvent-evaporation or annealing	N/A		200 (native)	N/A
							Dissolving in solution	SS		164-221	490-676
								TC-SS		≥ 200 (native)	450-510
	1[28]	844	1.39	4214	5947	0.7	Shearing	SS		N/A	499-626
	1[29]	988	1.20	3795	8702	0.4		SS		N/A	855
	1[24]	611	1.04	3284	3949	0.8		CL-SS		134	393
								CL-BCPB-SS		157	460
	1[25]	689	1.02	3197	5226	0.6	Solvent-evaporation	N/A		147 (native)	N/A
							Shearing	SS		N/A	416-492
	1[30]	590	1.09	3059	3984	0.8	N/A	SS		140	410
	1[29]	562	1.20	2968	3713	0.8	Shearing	SS or BCPB-SS		135-254	411-771

BCP properties							Self-assembly conditions			Optical characteristics	
**Name**[a]	**Articles (#)**	**M_n**[b] **(kDa)**	**PDI**[c]	**DP_1**[d]	**DP_2**[d]	$\frac{DP_1}{DP_2}$[d]	**Formation method**[e]	**Tuning method**[f]	**Solvent**[g]	***d***[h] **(nm)**	**λ_{max}**[i] **(nm)**
	1[28]	657	1.30	2965	5112	0.6		SS		153-178	425-517
								HPB-SS		158-181	476-542
	1[31]	741	1.12	2727	6709	0.4	Solvent-evaporation	N/A		≥ 106 (native)	305 (native)-675
	1[28]	517	1.31	2581	3643	0.7	Shearing	SS		≥ 116	≤ 427
								HPB-SS		N/A	407-485
	1[25]	527	1.02	2448	4008	0.6	Solvent-evaporation	N/A		118 (native)	N/A
							Shearing	SS		N/A	413-430
	2[32]	601	1.09	2372	5197	0.5	Dissolving in solution	SS		N/A	345-793
								TC-SS		134-292	377-836
	1[33]	418	N/A	2016	3053	0.7	Annealing	HPB		N/A	375-450
	1[34]	391	1.02	1863	2892	0.6		HPB		N/A	336-617
	1[35]	274	1.08	1315	2011	0.7		HPB		160	468
PS-*b*-PE/P	3[36]	800	1.04	3841	5703	0.7	Annealing	N/A	Toluene	200 (native)	545
								ND		≥ 180 (native)	UV-622
	1[37]	831	N/A	3601	6501	0.6		ND		≥ 180 (native)	UV-575
PS-*b*-P4VP	3[38]	288	1.23	2286	471	4.9	Annealing	SMC	Chloroform	140	N/A
							Solvent-evaporation	N/A		70 (native)	350
								SMC		97-140	370-470
							Annealing	SMC-TC		117-190	≤ 530
PS-*b*-P*t*BMA	1[39]	680	1.12	3044	2553	1.2	Dissolving in solution	SS-ND	Toluene; Acrylate monomer	143-180	428-543
	1[40]	N/A	N/A	N/A	N/A	N/A	Shearing-annealing	SS		180	532
PS-*b*-PMAA	1[41]	596	N/A	4801	1116	4.3	Annealing	SMC	THF	175	520
								SMC-TC		N/A	571-579
PS-*b*-PMMA†	1[42]	328	1.44	1709	1743	1.0	Dissolving in solution	SS	Styrene	120	483
P(E-*co*-P)-*b*-P(E-*co*-N)‡	1[43]	576	1.13	N/A	N/A	N/A	Annealing	N/A	Toluene	90 (native)	268
								HPB		123-150	335-448
P(MCP-*co*-VTM)-*b*-P(E-*co*-N) ‡	1[43]	451	1.41	N/A	N/A	N/A	Annealing	N/A	Toluene	170	470
LLDPE-*b*-ULDPE§	1[44]	68	2.04	N/A	N/A	N/A	Melting	HPB-SS	N/A	160 (native)-205	428 (native)-503
		47	2.07	N/A	N/A	N/A		N/A		140	369
PS-*b*-PSM‖	1[45]	1600	1.39	11234	2007	5.6	Solvent-evaporation	N/A	*o*-Xylene;	292	625

BCP properties							Self-assembly conditions			Optical characteristics	
**Name[a]**	**Articles (#)**	**M_n[b] (kDa)**	**PDI[c]**	**DP_1[d]**	**DP_2[d]**	**$\frac{DP_1}{DP_2}$[d]**	**Formation method[e]**	**Tuning method[f]**	**Solvent[g]**	***d*[h] (nm)**	**λ_{max}[i] (nm)**
		1400	1.39	8341	2560	3.3		N/A	Toluene	235	460
		1100	1.63	6554	1837	3.6		N/A		222	480
		970	1.60	5419	2007	2.7		N/A		257	550
		700	1.63	2880	2007	1.4		N/A		178	430
PTEPM-*b*-PSMA†	1[46]	237	1.37	666	553	1.2	Annealing	N/A	THF	127	365
								SS		145	458
								ND		126	365
								ND-SS		173	591
	1[46]	139	1.25	390	353	1.1		N/A		109	N/A
								SS		N/A	368
								ND		102	N/A
								ND-SS		159	568
PI-*b*-P4MS	1[47]	845	1.48	9118	5364	1.7	Annealing	N/A	THF; Chloroform	185	N/A
	1[47]	821	1.34	7574	4951	1.5		N/A		185-196	≥ 580
PS-*b*-PHEMA	1[48]	216	1.14	1455	488	3.0	Annealing	HPB*	THF	N/A	N/A
PBD-*b*-PEO	1[49]	66	1.03	627	731	0.9	Annealing	N/A	THF	78 (native)	N/A
								SS		122-185	360-495

† Prepared with RAFT Polymerization.

‡ Prepared with Living Olefin Polymerization.

§ Prepared with Coordinative Chain Transfer Polymerization.

|| Prepared with Reversible-Deactivation Radical Polymerization.

Table S6: Linear block copolymers for photonic multilayer particles.

BCP properties							Self-assembly conditions			Optical characteristics	
Name[a]	Articles (#)	M_n[b] (kDa)	PDI[c]	DP_1[d]	DP_2[d]	$\frac{DP_1}{DP_2}$[d]	Formation method[e]	Tuning method[f]	Solvent[g]	*d*[h] (nm)	λ_{max}[i] (nm)
PS-*b*-P2VP	1[50]	450	1.18	2400	1902	1.3	Solvent-evaporation	SS	Chloroform	N/A	565
								BCPB-SS			591
		536	1.14	2304	2815	0.8		SS		N/A	621
	2[50-51]	428	1.19-1.29	2045	2045	1.0		BCPB-SS		N/A	510-548
								SS		≥ 139	422-633
								SMC-SS		166-219	512-606
								SMC-HPB-SS		N/A	806
	2[51-52]	265	1.06-1.09	1277	1255	1.0	Solvent-evaporation or annealing	N/A		99/97 (native)	320 (native)
							Solvent-evaporation	SMC-HPB-SS		N/A	561
								SMC-SS		145-166	475-529
							Annealing	SS		≥ 160	472-700
								CL-SS		N/A	453-668
	1[9e]	199	N/A	979	923	1.1	Solvent-evaporation	SS		N/A	N/A
	1[9e, 12]	142	N/A	720	632	1.1		SS		N/A	N/A
	1[9e, 12]	114	N/A	547	542	1.0		SS		N/A	N/A
PS-*b*-P4VP	1[53]	427	1.15	3361	732	4.6	Solvent-evaporation	SMC	Chloroform	166	550
	1[51]	398	N/A	1901	1902	1.0		SS		N/A	UV
								SMC-SS			N/A

Table S7: Brush block copolymers for photonic multilayer films.

BCP properties									Self-assembly conditions			Optical characteristics	
Name^a	Articles (#)	M_n^b (kDa)	PDI^c	DP_1^d	M_n (SC_1) (kDa)^b	DP_2^d	M_n (SC_2) (kDa)^b	$\frac{DP_1}{DP_2}^d$	Formation method^e	Tuning method^f	Solvent^g	d^h (nm)	λ_{max}^i (nm)
P(PS)-*b*-P(PDLLA)#	1[54]	2400	N/A	420	3.2	210	2.6	2.0	Annealing	CGD	N/A	153	N/A
		2000			3.2		2.6					163	
		1700	N/A	210	3.0	210	2.9	1.0		CSC-CGD		105	
		1200			3.0		1.4					100	
		2400			5.7		3.6					126	
P(PLLA)-*b*-P(PVP)##	1[55]	2070	1.16	190	4.7	200	5.4	1.0	Solvent-evaporation	N/A	N/A	202	N/A
P(I-PS)-*b*-P(I-PDLLA)	3[56]	1080-3320	1.07-1.29	98-309	5.7	84-252	6.1	1.2	Solvent-evaporation	CBBDP-CPS	DCM; THF	N/A	227-576
		1080-3320		98-309		84-252		1.2	Solvent-evaporation and annealing	CBBDP-CPS		N/A	254-874
		1080-6640	1.07-1.58	98-624		84-497		1.2-1.4	Thermal annealing	CBBDP		N/A	234-1311
		964	1.02	155	3.4	143	2.8	1.1	Annealing	HPB		128 (native)-197	391 (native)-530
		1392	1.01	217		209		1.0		HPB		143 (native)-227	442 (native)-685
		1495	1.02	230		230		1.0		HPB		≤ 290	389-874
		1730	1.02	267		267		1.0		HPB		195 (native)-346	574 (native)-1042
		2033	1.04	320		320		1.0		HPB		227 (native)-309	695 (native)-961
		2520	1.05	401		401		1.0		HPB		275 (native)-426	829 (native)-1286
		2882	1.05	460		460		1.0		HPB		305 (native)-485	921 (native)-1403
		520-1560	1.03-1.31	88-251	4.4	55-132	3.4	1.6-2.1		CBBDP		≥ 79	310-653
P(I-PS)-*b*-P(N-PDLLA)	1[56c]	524-2130	1.03-1.14	88-367	4.4	42-158	3.3	2.1-2.4	Annealing	CBBDP	N/A	≥ 63	293-568
P(N-PS)-*b*-P(I-PDLLA)	1[56c]	613-2600	1.03-1.08	109-485	3.6	63-243	3.4	1.7-2.1	Annealing	CBBDP	N/A	≥ 89	312-479
P(N-PS)-*b*-P(N-PDLLA)	4[56c, 57]	414-2300	1.02-1.04	74-441	3.6	45-212	3.3	1.6-2.1	Annealing	CBBDP	Toluene	≥ 73	317-407
		610	1.03	196	4.5	210	4.2	0.9	Dissolving in solution and shearing	SH		153-161	467-493
		592	1.05	194	4.5	222	4.2	0.9	Dissolving in solution	SS		N/A	N/A
P(I-PS)-*b*-P(I-P*t*BOS)	1[58]	1583-2704	1.07-1.13	307-566	2.7	287-507	3.0	1.1-1.2	Annealing	CBBDP	N/A	N/A	440-640
P(I-PS)-*b*-P(I-PHS)	1[59]	1215	1.12	248	2.6	240	2.4	1.0	Solvent-evaporation and annealing	HPB	1,4-Dioxane	133	N/A
		1400	1.09	308		252		1.2		HPB		129-183	429-541
		1630	1.18	349		304		1.1		HPB		150-207	497-640

BCP properties									Self-assembly conditions			Optical characteristics	
Name[a]	Articles (#)	M_n[b] (kDa)	PDI[c]	DP_1[d]	M_n (SC_1) (kDa)[b]	DP_2[d]	M_n (SC_2) (kDa)[b]	$\frac{DP_1}{DP_2}$[d]	Formation method[e]	Tuning method[f]	Solvent[g]	*d*[h] (nm)	λ_{max}[i] (nm)
		2051	1.18	435		387		1.1		HPB		178-230	≤ 680
P(I-PS)-*b*-P(I-PMMA)	1[60]	1468	1.20	234	3.0	207	3.7	1.1	Solvent-evaporation	N/A	N/A	129	380
P(I-PS)-*b*-P(N-PMMA)	1[61]	1774-2258	1.14-1.18	330-409	2.7	328-427	2.8	1.0	Solvent-evaporation and annealing	CBBDP	THF	156-197	479-556
P(I-PS)-b-P(N-P*t*BMA)	1[61]	1442-3002	1.12-1.17	235-495	2.7	233-481	3.5	1.0	Solvent-evaporation and annealing	CBBDP	THF	110-256	387-690
P(I-PS)-*b*-P(I-P*t*BA)	2[62]	242-595	1.30-2.10	80-150	3.2	80-150	3.6	1.0	Solvent-evaporation and annealing	CBBDP	DCM	85-155	294-480
		214	1.30	N/A	N/A	N/A	N/A	N/A	Annealing	TC		108 (native)-160	360 (native)-450
		304	2.00	N/A	N/A	N/A	N/A	N/A		TC		N/A	360 (native)-412
P(I-PS)-*b*-P(N-PEO)	1[63]	890	1.39	127	3.5	221	2.0	0.6	Solvent-evaporation	ND	THF	N/A	678
									Solvent-evaporation and annealing				718-805
		995	1.28	142		248		0.6	Solvent-evaporation	ND		120-185	458-662
									Solvent-evaporation and annealing			N/A	736
		1150	1.36	177		264		0.7	Solvent-evaporation	ND		223	796
		1330	1.49	190		332		0.6		ND		260	1009
P(I-P*t*BA)-*b*-P(N-PEO)	2[64]	1590-2760	1.05-1.15	128-233	8.2	108-169	5.0	1.2-1.4	Solvent-evaporation and annealing	CBBDP	THF	91-196	≤ 357
		2150	1.08	172		148		1.2		ND		135-137	380-398
		2760	1.05	233		169		1.4		ND		N/A	391-395
		2150	1.08	172		148		1.2		CL		133 (native)-185	≤ 463
		2760	1.05	233		169		1.4		CL		≥ 196 (native)	≤ 552
P(N-P*n*BA)-*b*-P(N-PEO)	1[65]	192-401	1.31-1.53	175-275	3.1	175-275	2.7	1.0	Annealing	CBBDP	N/A	163-249	400-622
P(I-PHI)-*b*-P(I-P4PBI)	2[66]	1400-5122	1.08-1.39	115-419	6.5	104-382	5.6	1.0-1.1	Solvent-evaporation	CBBDP	DCM	N/A	334-1120
		1400	1.08	115		104		1.1		BCPB		N/A	410-701
P(I-AW)-*b*-P(I-BnW)	1[67]	457-1136	1.05-1.24	326-776	0.8	322-818	0.6	0.9-1.0	Solvent-evaporation	CBBDP	DCM	N/A	330-888
		457-1136	1.05-1.24	326-776	0.8	322-818	0.6	0.9-1.0	Solvent-evaporation and annealing	CBBDP		N/A	440-1223
P(I-DDW)-*b*-P(I-BnW)	1[68]	488-841	1.04-1.12	N/A	N/A	N/A	N/A	N/A	Annealing	CBBDP	N/A	N/A	343-480
P(I-DDW)-*b*-P(I-FBnW)	1[68]	564-1040	1.03-1.09	N/A	N/A	N/A	N/A	N/A	Annealing	CBBDP	N/A	N/A	410-610

BCP properties									Self-assembly conditions			Optical characteristics	
Name[a]	Articles (#)	M_n[b] (kDa)	PDI[c]	DP_1[d]	M_n (SC_1) (kDa)[b]	DP_2[d]	M_n (SC_2) (kDa)[b]	$\frac{DP_1}{DP_2}$[d]	Formation method[e]	Tuning method[f]	Solvent[g]	*d*[h] (nm)	λ_{max}[i] (nm)
									Shearing			N/A	387-737
P(I-AM)-*b*-P(I-BzM)	1[69]	388-1169	1.10-1.40	N/A	N/A	N/A	N/A	N/A	Solvent-evaporation	CBBDP	THF	110-523	328-1231
P(I-AM-*r*-I-DMA)-*b*-P(I-BW)	1[70]	227	1.37	N/A	N/A	N/A	N/A	N/A	Solvent-evaporation and annealing	CL-SS	N/A	≥ 110	461 (native)-705
P(I-POSS)-*b*-P(N-PBzMA)	1[71]	805-1643	1.06-1.30	225-837	1.2	98-199	5.4	2.3-9.1	Solvent-evaporation and annealing	CBBDP	THF	138-290	391-841
P(N-PDLLA)-*b*-P(N-PDMS)	2[57b, 72]	1930	1.09	200	5.1	200	6.2	1.0	Solvent-evaporation / shearing	TC-SH	THF	204-229	403-626
P(I-PDLLA)-*b*-P(I-P*n*BA)	1[73]	1770	1.09	200	4.7	200	4.0	1.0	Solvent-evaporation	SS	N/A	N/A	N/A
P(N-PS)-*b*-P(N-PDMS)	1[74]	3300-7100	1.43-1.58	N/A	4.7	N/A	4.9	N/A	Solvent-evaporation	CBBDP	Chloroform	N/A	483-625
P(I-PS)-*b*-P(I-PLLA)	2[75]	763	1.02	135	2.5	152	2.8	0.9	Solvent-evaporation and annealing	CBBDP	DCM	138	N/A
		1316	1.16	N/A	9.8	N/A	9.7	N/A		TC	THF	68-208	650

[#] P(PS)-*b*-P(PDLLA) represents poly(styrene)-*b*-poly(lactide) and is prepared with a combination of RAFT, ATRP, and ROP. [##] P(PLLA)-*b*-P(PVP) represents poly(lactide)-*b*-poly(N-vinylpyrrolidone) and is prepared with a combination of RAFT and ROP. Here poly(styrene), poly(lactide), and poly(N-vinylpyrrolidone) refer to the side chains, however, the BBCP backbone is composed of vinyl monomers instead of being based upon norbornene.

Table S8: Brush block copolymers for photonic multilayer particles.

BCP properties									Self-assembly conditions			Optical characteristics	
Name[a]	Articles (#)	M_n[b] (kDa)	PDI[c]	DP_1[d]	M_n (SC_1) (kDa)[b]	DP_2[d]	M_n (SC_2) (kDa)[b]	$\frac{DP_1}{DP_2}$[d]	Formation method[e]	Tuning method[f]	Solvent[g]	*d*[h] (nm)	λ_{max}[i] (nm)
P(N-PS)-*b*-P(N-PDMS)	1[74]	3300-7100	1.43-1.58	N/A	4.7	N/A	4.9	N/A	Solvent-evaporation	CBBDP	Chloroform	N/A	489-645
		3300-4100								BCPB			521-614
		3300-7100								SS			501-683
		3300	1.58							ND			513
P(I-AW)-*b*-P(I-BnW)	1[76]	415-969	1.11-1.20	308-703	0.8	277-668	0.6	0.9-1.1	Solvent-evaporation	CBBDP	DCM	153-298	369-659
P(I-PS)-*b*-P(I-PLLA)	1[75a]	763	1.02	135	2.5	152	2.8	0.9	Solvent-evaporation	CBBDP	Benzene	N/A	N/A
P(I-PS)-*b*-P(N-PDMS)	1[77]	1050	1.36	130	4.4	177	4.7	0.7	Solvent-evaporation	HPB	Chloroform	154-186	472-541
		2000	1.21	220		300						N/A	647-759
		1050	1.36	130		177				MF			427-520
P(I-PS)-*b*-P(N-PDMS) (crosslinked with PDMS-based bis-benzophenone for P(N-PDMS))		1050	1.21	130		177			Solvent-evaporation and crosslinking	CL-MF			402-530
		2000	1.36	220		300							596-685

Table S9: Brush block copolymers for photonic porous particles.

BCP properties									Self-assembly conditions			Optical characteristics	
**Name[a]**	**Articles (#)**	**M_n[b] (kDa)**	**PDI[c]**	**DP_1[d]**	**M_n (SC_1) (kDa)[b]**	**DP_2[d]**	**M_n (SC_2) (kDa)[b]**	**$\frac{DP_1}{DP_2}$[d]**	**Formation method[e]**	**Tuning method[f]**	**Solvent[g]**	**2ξ[h] (nm)**	**λ_{max}[i] (nm)**
P(I-PS)-*b*-P(I-PEO) (with FeCp in PEO)	1[78]	355	1.08	36	5.3	42	4.6	0.9	Solvent-evaporation	RR-H_2O_2	Toluene	144-301	420-650
		355	1.08	36		42		0.9		RR-Salt		N/A	385-450
		258	1.07	26		30		0.9		RR-H_2O_2		N/A	UV-450
		403	1.09	44		51		0.9		RR-H_2O_2		N/A	622-721
P(I-PS)-*b*-P(I-PEO)		416	1.07	42	5.3	48	4.6	0.9		RR-H_2O_2		N/A	635-650
		293-299	1.08-1.09	27-46	4.1	27-46	4.6	0.6-1.7		CVF-PEO		N/A	413-585
P(I-PS)-*b*-P(N-PEO)	1[79]	274	N/A	N/A	4.0	N/A	4.0	N/A	Solvent-evaporation	CHGST	Toluene	133-193	417-652
										CHGST-Dry		N/A	392-505
P(I-PS)-*b*-P(N-PEO)	1[80]	371-626	1.07-1.53	43-74	4.3	46-77	4.0	0.9-1.0	Solvent-evaporation	CBBDP	Toluene	160-390	≥ 416
		475	1.28	54		61		0.9		CSET		N/A	428-645
P(I-PS)-*b*-P(I-PEO) (with TPE in PS)	1[81]	204-293	1.17-1.31	28-42	4.4	27-39	4.6	1.0-1.1	Solvent-evaporation	CBBDP	Toluene	195-301	482-677
P(I-PS)-*b*-P(I-PEO) (with TPE in PEO)		161-187	1.16-1.26	22-27		21-26		1.0				154-296	423-665
P(N-PDMS)-*b*-P(N-PEO) (crosslinked with PDMS-based bis-benzophenone for P(N-PDMS))	1[82]	677	1.18	76	4.7	76	4.0	1.0	Solvent-evaporation and crosslinking	CL-SS	Toluene	160-178	UV-682
		408	1.10	46		46		1.0				N/A	477-551
P(N-PDMS)-*b*-P(N-PEO)		408	1.10	46		46		1.0	Solvent-evaporation	SS		N/A	474-560
		439	1.13	49		49		1.0		CSET		N/A	484-605
		297-677	1.10-1.20	33-76		33-76		1.0		CBBDP		N/A	458-756

Table S10: Refractive index of LBCP blocks and BBCP sidechains.

Name	Refractive index
PS	1.59[3e, 11]
P2VP	1.62[3e, 11]
PI	1.51[27a, 31]
PE/P	1.48[36a]
P4VP	1.60[83]
P*t*BMA	1.46[39]
PMAA	1.45[41]
PMMA	1.49[84] (1.45 - 1.51) [85]
P(E-*co*-P)	1.47[43]
P(E-*co*-N)	1.52[43]
P(MCP-*co*-VTM)	1.50[43]
LLDPE	1.51[44]
ULDPE	1.47[44]
PSM	1.44 (based on monomer, from sigmaaldrich.com)
PTEPM	1.46[86]
PSMA	1.50[87]
P4MS	1.57[47]
PHEMA	1.51[88]
PBD	1.50[49]
PEO	1.46[49]
PDLLA	1.44 - 1.48[89]
PLLA	1.47[90]
PVP	1.51 - 1.56[91]
P*t*BOS	1.52 (based on monomer, from sigmaaldrich.com)
PHS	1.61[59]
P*t*BA	1.47[92]
P*n*BA	1.46[65]
PHI	1.42 (based on monomer, from sigmaaldrich.com)
P4PBI	1.51 (based on monomer, from sigmaaldrich.com)
AW	Not reported
BnW	Not reported
DDW	Not reported
FBnW	Not reported
AM	Not reported
BzM	Not reported
AM-*r*-I-DMA	Not reported
BW	Not reported
POSS	Not reported
PBzMA	1.57[93]
PDMS	1.41-1.44[74]

Section S4. Statistics of linear and brush block copolymers to indicate the relationship between domain spacing (*d*) and degree of polymerization (DP)

The d and DP were either taken from the following source articles or, if not directly available, estimated from other reported parameters or extracted from the associated figures.

BBCPs:
P(I-AM)-*b*-P(I-BzM) [69]
P(I-PS)-*b*-P(I-PDLLA) [56b, 94]
P(I-PS)-*b*-P(N-P*t*BMA) [61]

LBCPs:
PS-*b*-P2VP [3a, 3d, 3e, 8-9, 9d, 11, 13, 18i, 19, 21, 52, 95]
PS-*b*-PI [25, 27a, 31, 35, 96]
PS-*b*-PIBOH (polystyrene-*b*-polyhydroxy isobutylene) [97]

References

[1] a) J. Sun, B. Bhushan, J. Tong, *RSC Adv.* **2013**, *3*, 14862; b) H. Fudouzi, *Science and Technology of Advanced Materials* **2011**, *12*, 064704; c) A. Richel, N. P. Johnson, D. W. McComb, *Appl. Phys. Lett.* **2000**, *76*, 1816; d) H. Fudouzi, *J. Colloid Interf. Sci.* **2004**, *275*, 277.

[2] E. L. Lin, W. L. Hsu, Y. W. Chiang, *ACS Nano* **2018**, *12*, 485.

[3] a) Y. Kang, J. J. Walish, T. Gorishnyy, E. L. Thomas, *Nat. Mater.* **2007**, *6*, 957; b) Y. Kang, *B. Kor. Chem. Soc.* **2008**, *29*, 2329; c) Y. Kang, *Macromol. Res.* **2012**, *20*, 1223; d) C. Kang, E. Kim, H. Baek, K. Hwang, D. Kwak, Y. Kang, E. L. Thomas, *J. Am. Chem. Soc.* **2009**, *131*, 7538; e) Y. Lu, H. Xia, G. Zhang, C. Wu, *J. Mater. Chem.* **2009**, *19*, 5952; f) Y. Lu, C. Meng, H. Xia, G. Zhang, C. Wu, *J. Mater. Chem. C* **2013**, *1*, 6107.

[4] O. B. Ayyub, J. W. Sekowski, T.-I. Yang, X. Zhang, R. M. Briber, P. Kofinas, *Biosens. Bioelectron.* **2011**, *28*, 349.

[5] T. Lee, J. W. Ju, W. Ryoo, in *Advanced Fabrication Technologies for Micro/Nano Optics and Photonics V, Vol. 8249* (Eds.: W. V. Schoenfeld, R. C. Rumpf, G. VonFreymann), **2012**.

[6] a) O. B. Ayyub, M. B. Ibrahim, R. M. Briber, P. Kofinas, *Biosens. Bioelectron.* **2013**, *46*, 124; b) O. B. Ayyub, M. B. Ibrahim, P. Kofinas, *Polymer* **2014**, *55*, 6227.

[7] H. Xia, J. Zhao, C. Meng, Y. Wu, Y. Lu, J. Wang, Y. Song, L. Jiang, G. Zhang, *Soft Matter* **2011**, *7*, 4156.

[8] H. Eoh, Y. Jung, C. Park, C. E. Lee, T. H. Park, H. S. Kang, S. Jeon, D. Y. Ryu, J. Huh, C. Park, *Adv. Funct. Mater.* **2021**, 2103697.

[9] a) J. J. Walish, Y. Kang, R. A. Mickiewicz, E. L. Thomas, *Adv. Mater.* **2009**, *21*, 3078; b) E. P. Chan, J. J. Walish, E. L. Thomas, C. M. Stafford, *Adv. Mater.* **2011**, *23*, 4702; c) Y. Fan, S. Tang, E. L. Thomas, B. D. Olsen, *ACS Nano* **2014**, *8*, 11467; d) Y. Fan, J. J. Walish, S. Tang, B. D. Olsen, E. L. Thomas, *Macromolecules* **2014**, *47*, 1130; e) J. Y. Lim, J. H. Yun, D. bin Jang, D. M. Shin, in *Photonic Fiber and Crystal Devices: Advances in Materials and Innovations in Device Applications Ix, Vol. 9586* (Eds.: S. Yin, R. Guo), **2015**; f) Y. N. Oh, S. Y. Lee, D. M. Shin, *Mol. Cryst. Liq. Cryst.* **2017**, *653*, 17; g) E. Kim, C. Kang, H. Baek, K. Hwang, D. Kwak, E. Lee, Y. Kang, E. L. Thomas, *Adv. Funct. Mater.* **2010**, *20*, 1728; h) D. Kim, K.-S. Hwang, J.-H. Kim, C. Lee, J.-Y. Lee, *ACS Appl. Polym. Mater.* **2021**, *3*, 2902.

[10] J. Y. Lim, J. B. Ju, D. M. Shin, *Mol. Cryst. Liq. Cryst.* **2017**, *650*, 110.

[11] A. Noro, Y. Tomita, Y. Shinohara, Y. Sageshima, J. J. Walish, Y. Matsushita, E. L. Thomas, *Macromolecules* **2014**, *47*, 4103.

[12] J. Y. Lim, D. M. Shin, *Mol. Cryst. Liq. Cryst.* **2017**, *654*, 214.

[13] A. Noro, Y. Tomita, Y. Matsushita, E. L. Thomas, *Macromolecules* **2016**, *49*, 8971.

[14] T. H. Park, S. Yu, S. H. Cho, H. S. Kang, Y. Kim, M. J. Kim, H. Eoh, C. Park, B. Jeong, S. W. Lee, D. Y. Ryu, J. Huh, C. Park, *NPG Asia Mater.* **2018**, *10*, 328.

[15] T. H. Park, H. Eoh, Y. Jung, G.-W. Lee, C. E. Lee, H. S. Kang, J. Lee, K.-B. Kim, D. Y. Ryu, S. Yu, C. Park, *Adv. Funct. Mater.* **2021**, 2008548.

[16] H. Eoh, H. S. Kang, M. J. Kim, M. Koo, T. H. Park, Y. Kim, H. Lim, D. Y. Ryu, E. Kim, J. Huh, Y. Kang, C. Park, *Adv. Funct. Mater.* **2019**, *29*, 1904055.

[17] H. S. Kang, S. W. Han, C. Park, S. W. Lee, H. Eoh, J. Baek, D. G. Shin, T. H. Park, J. Huh, H. Lee, D. E. Kim, D. Ryu, E. L. Thomas, W. G. Koh, C. Park, *Science Advances* **2020**, *6*, eabb5769

[18] a) K. Hwang, D. Kwak, C. Kang, D. Kim, Y. Ahn, Y. Kang, *Angew. Chem. Int. Ed.* **2011**, *50*, 6311; b) H. S. Lim, J.-H. Lee, J. J. Walish, E. L. Thomas, *ACS Nano* **2012**, *6*, 8933; c) Y. Jang, J. Chung, S. Lee, H. Lim, H. Baek, Y. Kang, *Polym. Bull.* **2013**, *70*, 593; d) S. E. Shin, S. Y. Kim, D. M. Shin, *Mol. Cryst. Liq. Cryst.* **2010**, *520*, 398; e) S. E. Shin, S. Y. Kim, D. M. Shin, *J. Nanosci. Nanotechnol.* **2011**, *11*, 4275; f) S. W. Lee, J. Shin, J. Shin, C. Shim, D. M. Shin, *Mol. Cryst. Liq. Cryst.* **2011**, *539*, 96/[436]; g) Y. Ahn, E. Kim, J. Hyon, C. Kang, Y. Kang, *Adv. Mater.* **2012**, *24*, OP127; h) S. E. Shin, S. Y. Kim, D. M. Shin, in *Organic Photonic Materials and Devices Xii, Vol. 7599* (Eds.: R. L. Nelson, F. Kajzar, T. Kaino), **2010**; i) J. J. Walish, Y. Fan, A. Centrone, E. L. Thomas, *Macromol. Rapid. Comm.* **2012**, *33*, 1504; j) J. Y. Lim, S. Y. Park, S. W. Lee, J. H. Yun, S. H. Choi, D. M. Shin, *J. Nanosci. Nanotechnol.* **2016**, *16*, 8407; k) E. Lee, J. Kim, J. Myung, Y. Kang, *Macromol. Res.* **2012**, *20*, 1219; l) A. Subramanian, N. Tiwale, G. Doerk, K. Kisslinger, C. Y. Nam, *ACS Appl. Mater. Interfaces* **2020**, *12*, 1444; m) R. M. Zhang, Z. Qiang, M. Z. Wang, *Adv. Funct. Mater.* **2020**, *31*, 2005819.

[19] T. Heuser, R. Merindol, S. Loescher, A. Klaus, A. Walther, *Adv. Mater.* **2017**, *29*, 1606842.

[20] a) H. J. Kim, D. M. Shin, in *Advanced Fabrication Technologies for Micro/Nano Optics and Photonics V, Vol. 8249* (Eds.: W. V. Schoenfeld, R. C. Rumpf, G. VonFreymann), **2012**; b) H. J. Kim, D. M. Shin, *Mol. Cryst. Liq. Cryst.* **2013**, *581*, 101; c) Y. B. Baek, S. H. Choi, D. M. Shin, *Mol. Cryst. Liq. Cryst.* **2014**, *600*, 9; d) S. N. Lee, Y. Bin Baek, D. M. Shin, *J. Nanosci. Nanotechnol.* **2014**, *14*, 6053; e) H. J. Kim, D. M. Shin, *J. Nanosci. Nanotechnol.* **2014**, *14*, 6235; f) Y. B. Baek, S. H. Choi, D. M. Shin, *J. Nanosci. Nanotechnol.* **2015**, *15*, 1624; g) Y. N. Oh, S. Y. Lee, D. M. Shin, *Mol. Cryst. Liq. Cryst.* **2017**, *659*, 59; h) I. H. Lee, D. M. Shin, *Mol. Cryst. Liq. Cryst.* **2019**, *685*, 64.

[21] H. S. Kang, J. Lee, S. M. Cho, T. H. Park, M. J. Kim, C. Park, S. W. Lee, K. L. Kim, D. Y. Ryu, J. Huh, E. L. Thomas, C. Park, *Adv. Mater.* **2017**, *29*, 1700084.

[22] T. J. Park, S. K. Hwang, S. Park, S. H. Cho, T. H. Park, B. Jeong, H. S. Kang, D. Y. Ryu, J. Huh, E. L. Thomas, C. Park, *ACS Nano* **2015**, *9*, 12158.

[23] Y. Kang, *B. Kor. Chem. Soc.* **2012**, *33*, 2847.

[24] O. O. Mykhaylyk, A. J. Parnell, A. Pryke, J. P. A. Fairclough, *Macromolecules* **2012**, *45*, 5260.

[25] Y. W. Chiang, C. Y. Chou, C. S. Wu, E. L. Lin, J. Yoon, E. L. Thomas, *Macromolecules* **2015**, *48*, 4004.

[26] A. M. Urbas, E. L. Thomas, H. Kriegs, G. Fytas, R. S. Penciu, L. N. Economou, *Phys. Rev. Lett.* **2003**, *90*, 108302.

[27] a) W. Lee, J. Yoon, E. L. Thomas, H. Lee, *Macromolecules* **2013**, *46*, 6528; b) W. M. Lee, J. S. Yoon, H. J. Lee, E. L. Thomas, *Macromolecules* **2007**, *40*, 6021; c) J. Yoon, W. Lee, E. L. Thomas, *Macromolecules* **2008**, *41*, 4582.

[28] A. J. Parnell, N. Tzokova, A. Pryke, J. R. Howse, O. O. Mykhaylyk, A. J. Ryan, P. Panine, J. P. A. Fairclough, *Phys. Chem. Chem. Phys.* **2011**, *13*, 3179.

[29] A. J. Parnell, A. Pryke, O. O. Mykhaylyk, J. R. Howse, A. M. Adawi, N. J. Terrill, J. P. A. Fairclough, *Soft Matter* **2011**, *7*, 3721.

[30] J. Yoon, W. Lee, E. L. Thomas, *Nano Lett.* **2006**, *6*, 2211.

[31] Y. W. Chiang, J. J. Chang, C. Y. Chou, C. S. Wu, E. L. Lin, E. L. Thomas, *Adv. Optical Mater.* **2015**, *3*, 1517.

[32] a) A. Matsushita, S. Okamoto, *Macromolecules* **2014**, *47*, 7169; b) A. Matsushita, S. Okamoto, *Polym. J.* **2015**, *47*, 385.

[33] S. H. Kim, K. S. Kim, K. Char, S. I. Yoo, B. H. Sohn, *Nanoscale* **2016**, *8*, 10823.

[34] A. Urbas, R. Sharp, Y. Fink, E. L. Thomas, M. Xenidou, L. J. Fetters, *Adv. Mater.* **2000**, *12*, 812.

[35] A. Urbas, Y. Fink, E. L. Thomas, *Macromolecules* **1999**, *32*, 4748.

[36] a) M. Bockstaller, R. Kolb, E. L. Thomas, *Adv. Mater.* **2001**, *13*, 1783; b) M. R. Bockstaller, Y. Lapetnikov, S. Margel, E. L. Thomas, *J. Am. Chem. Soc.* **2003**, *125*, 5276; c) M. R. Bockstaller, E. L. Thomas, *J. Phys. Chem. B* **2003**, *107*, 10017.

[37] M. R. Bockstaller, E. L. Thomas, *Phys. Rev. Lett.* **2004**, *93*, 166106.

[38] a) J. Ruokolainen, M. Saariaho, O. Ikkala, G. ten Brinke, E. L. Thomas, M. Torkkeli, R. Serimaa, *Macromolecules* **1999**, *32*, 1152; b) H. Kosonen, S. Valkama, J. Ruokolainen, M. Torkkeli, R. Serimaa, G. ten Brinke, O. Ikkala, *Eur. Phys. J. E* **2003**, *10*, 69; c) S. Valkama, H. Kosonen, J. Ruokolainen, T. Haatainen, M. Torkkeli, R. Serimaa, G. Ten Brinke, O. Ikkala, *Nat. Mater.* **2004**, *3*, 872.

[39] K. Tsuchiya, S. Nagayasu, S. Okamoto, T. Hayakawa, T. Hihara, K. Yamamoto, I. Takumi, S. Hara, H. Hasegawa, S. Akasaka, N. Kosikawa, *Opt. Express* **2008**, *16*, 5362.

[40] T. Yamanaka, S. Hara, T. Hirohata, *Opt. Express* **2011**, *19*, 24583.

[41] C. Osuji, C. Y. Chao, I. Bita, C. K. Ober, E. L. Thomas, *Adv. Funct. Mater.* **2002**, *12*, 753.

[42] R. Motokawa, T. Taniguchi, T. Kumada, Y. Iida, S. Aoyagi, Y. Sasaki, M. Kohri, K. Kishikawa, *Macromolecules* **2016**, *49*, 6041.

[43] J. Yoon, R. T. Mathers, G. W. Coates, E. L. Thomas, *Macromolecules* **2006**, *39*, 1913.

[44] P. D. Hustad, G. R. Marchand, E. I. Garcia-Meitin, P. L. Roberts, J. D. Weinhold, *Macromolecules* **2009**, *42*, 3788.

[45] J. K. D. Mapas, T. Thomay, A. N. Cartwright, J. Ilavsky, J. Rzayev, *Macromolecules* **2016**, *49*, 3733.

[46] Y. C. Huang, Y. Zheng, J. Pribyl, B. C. Benicewicz, *J. Mater. Chem. C* **2017**, *5*, 9873.

[47] M. Appold, E. Grune, H. Frey, M. Gallei, *ACS Appl. Mater. Interfaces* **2018**, *10*, 18202.

[48] M. Plank, F. Hartmann, B. Kuttich, T. Kraus, M. Gallei, *Eur. Polym. J.* **2020**, 110059.

[49] Y. F. Xu, R. J. Hickey, *Macromolecules* **2020**, *53*, 5711.

[50] Y. Yang, Y. Chen, Z. Hou, F. Li, M. Xu, Y. Liu, D. Tian, L. Zhang, J. Xu, J. Zhu, *ACS Nano* **2020**, *14*, 16057.

[51] Y. Yang, T. H. Kang, K. Wang, M. Ren, S. B. Chen, B. J. Xiong, J. P. Xu, L. B. Zhang, G. R. Yi, J. T. Zhu, *Small* **2020**, *16*, 2001315.

[52] Y. Yang, H. Kim, J. P. Xu, M. S. Hwang, D. Tian, K. Wang, L. B. Zhang, Y. G. Liao, H. G. Park, G. R. Yi, X. L. Xie, J. T. Zhu, *Adv. Mater.* **2018**, *30*, 1707344.

[53] G. Moriceau, C. Kilchoer, K. Djeghdi, C. Weder, U. Steiner, B. D. Wilts, I. Gunkel, *Macromol. Rapid. Comm.* **2021**, 2100522.

[54] J. Rzayev, *Macromolecules* **2009**, *42*, 2135.

[55] Y. H. Wang, R. Ren, J. Ling, W. L. Sun, Z. Q. Shen, *Polymer* **2018**, *138*, 378.

[56] a) B. R. Sveinbjoernsson, R. A. Weitekamp, G. M. Miyake, Y. Xia, H. A. Atwater, R. H. Grubbs, *P. Natl. Acad. Sci. USA* **2012**, *109*, 14332; b) R. J. Macfarlane, B. Kim, B. Lee, R. A. Weitekamp, C. M. Bates, S. F. Lee, A. B. Chang, K. T. Delaney, G. H. Fredrickson, H. A. Atwater, R. H. Grubbs, *J. Am. Chem. Soc.* **2014**, *136*, 17374; c) B. M. Boyle, J. L. Collins, T. E. Mensch, M. D. Ryan, B. S. Newell, G. M. Miyake, *Polym. Chem.* **2020**, *11*, 7147.

[57] a) M. A. Wade, D. Walsh, J. C. W. Lee, E. Kelley, K. Weigandt, D. Guironnet, S. A. Rogers, *Soft Matter* **2020**, *16*, 4919; b) B. B. Patel, Y. Chang, S. K. Park, S. Wang, J. Rosheck, K. Patel, D. Walsh, D. Guironnet, Y. Diao, *J. Polym. Sci.* **2021**, 1; c) T. Pan, B. B. Patel, D. J. Walsh, S. Dutta, D. Guironnet, Y. Diao, C. E. Sing, *Macromolecules* **2021**, *54*, 3620.

[58] Y. G. Yu, C. G. Chae, M. J. Kim, H. B. Seo, R. H. Grubbs, J. S. Lee, *Macromolecules* **2018**, *51*, 447.

[59] Y. G. Yu, C. Seo, C. G. Chae, H. B. Seo, M. J. Kim, Y. Kang, J. S. Lee, *Macromolecules* **2019**, *52*, 4349.

[60] M. J. Kim, Y. G. Yu, C. G. Chae, H. B. Seo, I. G. Bak, Y. Mallela, J. S. Lee, *Macromolecules* **2019**, *52*, 103.

[61] H. B. Seo, Y. G. Yu, C. G. Chae, M. J. Kim, J. S. Lee, *Polymer* **2019**, *177*, 241.

[62] a) Y. P. Qiao, Y. D. Zhao, X. Y. Yuan, Y. H. Zhao, L. X. Ren, *J. Mater. Sci.* **2018**, *53*, 16160; b) T. T. Guo, Y. Wang, Y. P. Qiao, X. Y. Yuan, Y. H. Zhao, L. X. Ren, *Polymer* **2020**, *194*, 122389.

[63] D. P. Song, C. Li, N. S. Colella, X. M. Lu, J. H. Lee, J. J. Watkins, *Adv. Optical Mater.* **2015**, *3*, 1169.

[64] a) D. P. Song, C. Li, W. H. Li, J. J. Watkins, *ACS Nano* **2016**, *10*, 1216; b) D. P. Song, G. Jacucci, F. Dundar, A. Naik, H. F. Fei, S. Vignolini, J. J. Watkins, *Macromolecules* **2018**, *51*, 2395.

[65] T. T. Guo, X. L. Yu, Y. H. Zhao, X. Y. Yuan, J. Y. Li, L. X. Ren, *Macromolecules* **2020**, *53*, 3602.

[66] a) G. M. Miyake, R. A. Weitekamp, V. A. Piunova, R. H. Grubbs, *J. Am. Chem. Soc.* **2012**, *134*, 14249; b) G. M. Miyake, V. A. Piunova, R. A. Weitekamp, R. H. Grubbs, *Angew. Chem. Int. Ed.* **2012**, *51*, 11246.

[67] V. A. Piunova, G. M. Miyake, C. S. Daeffler, R. A. Weitekamp, R. H. Grubbs, *J. Am. Chem. Soc.* **2013**, *135*, 15609.

[68] B. M. Boyle, T. A. French, R. M. Pearson, B. G. McCarthy, G. M. Miyake, *ACS Nano* **2017**, *11*, 3052.

[69] T. Z. Zhang, J. X. Yang, X. L. Yu, Y. S. Li, X. Y. Yuan, Y. H. Zhao, D. Lyu, Y. F. Men, K. Zhang, L. X. Ren, *Polym. Chem.* **2019**, *10*, 1519.

[70] Y. D. Zhao, T. T. Guo, J. X. Yang, Y. S. Li, X. Y. Yuan, Y. H. Zhao, L. X. Ren, *React. Funct. Polym.* **2019**, *139*, 162.

[71] C. G. Chae, Y. G. Yu, M. J. Kim, R. H. Grubbs, J. S. Lee, *Macromolecules* **2018**, *51*, 3458.

[72] B. B. Patel, D. J. Walsh, D. H. Kim, J. Kwok, B. Lee, D. Guironnet, Y. Diao, *Science Advances* **2020**, *6*, eaaz7202.

[73] Y. Xia, B. D. Olsen, J. A. Kornfield, R. H. Grubbs, *J. Am. Chem. Soc.* **2009**, *131*, 18525.

[74] D. P. Song, T. H. H. Zhao, G. Guidetti, S. Vignolini, R. M. Parker, *ACS Nano* **2019**, *13*, 1764.

[75] a) E. J. Kim, J. J. Shin, T. Do, G. S. Lee, J. Park, V. Thapar, J. Choi, J. Bang, G.-R. Yi, S.-M. Hur, J. G. Kim, B. J. Kim, *ACS Nano* **2021**, *15*, 5513; b) C. Liang, J. Hou, Y. Li, D. Liu, J. Li, X. Cui, Q. Duan, *Opt. Mater.* **2021**, *111*, 110590.

[76] Q. He, K. H. Ku, H. Vijayamohanan, B. J. Kim, T. M. Swager, *J. Am. Chem. Soc.* **2020**, *142*, 10424.

[77] Y. Dong, Z. Ma, D.-P. Song, G. Ma, Y. Li, *ACS Nano* **2021**, *15*, 8770.

[78] Y.-L. Li, X. Chen, H.-K. Geng, Y. Dong, B. Wang, Z. Ma, L. Pan, G.-Q. Ma, D.-P. Song, Y.-S. Li, *Angew. Chem. Int. Ed.* **2020**, *60*, 3647.

[79] T. H. Zhao, G. Jacucci, X. Chen, D.-P. Song, S. Vignolini, R. M. Parker, *Adv. Mater.* **2020**, *32*, 2002681.

[80] X. Chen, X. Yang, D.-P. Song, Y.-F. Men, Y. Li, *Macromolecules* **2021**, *54*, 3668.

[81] Q.-J. Liu, Y. Li, J.-C. Xu, H.-F. Lu, Y. Li, D.-P. Song, *ACS Nano* **2021**, *15*, 5534.

[82] X. Li, B. Wang, Q.-J. Liu, R. Zhao, D.-P. Song, Y. Li, *Langmuir* **2021**, *22*, 6744.

[83] L. C. Cesteros, J. R. Isasi, I. Katime, *Macromolecules* **1994**, *27*, 7887.

[84] a) G. Beadie, M. Brindza, R. A. Flynn, A. Rosenberg, J. S. Shirk, *Appl. Optics.* **2015**, *54*, F139; b) N. Tanio, T. Nakanishi, *Polym. J.* **2006**, *38*, 814.

[85] a) D. J. Chapman, D. E. Eakins, D. M. Williamson, W. Proud, *AIP Conference Proceedings* **2012**, *1426*, 442; b) A. K. Othayoth, B. Srinivas, K. Murugan, K. Muralidharan, *Opt. Mater.* **2020**, *104*, 109841; c) R. Kallweit, J. P. Biersack, *Radiat. Eff. Defect. S.* **1991**, *116*, 29.

[86] D. Parisi, J. Ruiz-Franco, YingboRuan, C. YiangLiu, B. Loppinet, E. Zaccarelli, D. Vlassopoulos, *Phys. Fluids.* **2020**, *32*, 127101.

[87] G. N. Smith, M. J. Derry, J. E. Hallett, J. R. Lovett, O. O. Mykhaylyk, T. J. Neal, S. Prévost, S. P. Armes, *P. Roy. Soc. A* **2019**, *475*, 20180763.

[88] a) A. De Girolamo Del Mauro, A. I. Grimaldi, V. La Ferrara, E. Massera, M. L. Miglietta, T. Polichetti, G. Di Francia, *J. Sensors* **2009**, *2009*, 703206; b) X. Xu, A. V. Goponenko, S. A. Asher, *J. Am. Chem. Soc.* **2008**, *130*, 3113.

[89] a) A. Gierej, A. Filipkowski, D. Pysz, R. Buczyński, M. Vagenende, P. Dubruel, H. Thienpont, T. Geernaert, F. Berghmans, *J. Lightwave. Technol.* **2020**, *38*, 1905; b) A. Gierej, M. Vagenende, A. Filipkowski, B. Siwicki, R. Buczynski, H. Thienpont, S. V. Vlierberghe, T. Geernaert, P. Dubruel, F. Berghmans, *J. Lightwave. Technol.* **2019**, *37*, 1916.

[90] R. Fu, W. Luo, R. Nazempour, D. Tan, H. Ding, K. Zhang, L. Yin, J. Guan, X. Sheng, *Adv. Optical Mater.* **2018**, *6*, 1700941.

[91] a) Y. Pei, F. Yao, P. Ni, X. Sun, *J. Mod. Optic.* **2010**, *57*, 872; b) M. Guettari, A. Belaidi, S. Abel, T. Tajouri, *J. Solution. Chem.* **2017**, *46*, 1404; c) M. Guettari, A. Gharbi, *J. Macromol. Sci. B* **2010**, *49*, 592; d) M. Morisawa, H. Yamaoka, Y. Suzuki, in *2016 IEEE SENSORS*, **2016**, pp. 1.

[92] T. Wu, P. Gong, I. Szleifer, P. Vlček, V. Šubr, J. Genzer, *Macromolecules* **2007**, *40*, 8756.

[93] D. W. Van Krevelen, K. Te Nijenhuis, in *Properties of Polymers (Fourth Edition)* (Eds.: D. W. Van Krevelen, K. Te Nijenhuis), Elsevier, Amsterdam, **2009**, pp. 287.

[94] a) L. Jiang, D. Nykypanchuk, V. J. Pastore, J. Rzayev, *Macromolecules* **2019**, *52*, 8217; b) T.-P. Lin, A. B. Chang, S.-X. Luo, H.-Y. Chen, B. Lee, R. H. Grubbs, *ACS Nano* **2017**, *11*, 11632; c) W. Gu, J. Huh, S. W. Hong, B. R. Sveinbjornsson, C. Park, R. H. Grubbs, T. P. Russell, *ACS Nano* **2013**, *7*, 2551.

[95] a) C.-C. Wang, K.-H. Wu, C.-T. Lo, *Macromolecules* **2019**, *52*, 3210; b) Y. Kim, D. Yong, W. Lee, H. Ahn, J. H. Kim, J. U. Kim, D. Y. Ryu, *Macromolecules* **2019**, *52*, 8672; c) A. Alvarez-Fernandez, K. Aissou, G. Pécastaings, G. Hadziioannou, G. Fleury, V. Ponsinet, *Nanoscale Adv.* **2019**, *1*, 849; d) B. V. K. J. Schmidt, C. X. Wang, S. Kraemer, L. A. Connal, D. Klinger, *Polym. Chem.* **2018**, *9*, 1638; e) Y. Kim, D. Yong, W. Lee, S. Jo, H. Ahn, J. U. Kim, D. Y. Ryu, *Macromolecules* **2018**, *51*, 8550; f) N. Yan, Y. Zhang, Y. He, Y. Zhu, W. Jiang, *Macromolecules* **2017**, *50*, 6771; g) H. Wakayama, H. Yonekura, *Macromol. Res.* **2017**, *25*, 201; h) Y.-S. Sun, W.-H. Huang, C.-F. Lin, S.-L. Cheng, *Langmuir* **2017**, *33*, 2003; i) J. Lee, J. Kwak, C. Choi, S. H. Han, J. K. Kim, *Macromolecules* **2017**, *50*, 9373; j) S. H. Kim, K. Char, S. I. Yoo, B.-H. Sohn, *Adv. Funct. Mater.* **2017**, *27*, 1606715; k) Y.-S. Lu, C.-Y. Yu, Y.-C. Lin, S.-W. Kuo, *Soft Matter* **2016**, *12*, 2288; l) C. G. Chandaluri, G. Pelossof, R. Tel-Vered, R. Shenhar, I. Willner, *ACS Appl. Mater. Interfaces* **2016**, *8*, 1440; m) C. G. Arges, Y. Kambe, H. S. Suh, L. E. Ocola, P. F. Nealey, *Chem. Mater.* **2016**, *28*, 1377; n) C.-T. Lo, M.-H. Li, W.-T. Lin, *J. Chem. Phys.* **2015**, *142*, 184903; o) J. Wernecke, H. Okuda, H. Ogawa, F. Siewert, M. Krumrey, *Macromolecules* **2014**, *47*, 5719; p) A. Stenbock-Fermor, A. W. Knoll, A. Böker, L.

Tsarkova, *Macromolecules* **2014**, *47*, 3059; q) I. Davidi, D. Patra, D. Hermida-Merino, G. Portale, V. M. Rotello, U. Raviv, R. Shenhar, *Macromolecules* **2014**, *47*, 5774; r) H. Wakayama, H. Yonekura, Y. Kawai, *ACS Macro Lett.* **2013**, *2*, 284; s) C.-T. Lo, W.-T. Lin, *J. Phys. Chem. B* **2013**, *117*, 5261; t) C. Liedel, C. W. Pester, M. Ruppel, C. Lewin, M. J. Pavan, V. S. Urban, R. Shenhar, P. Bösecke, A. Böker, *ACS Macro Lett.* **2013**, *2*, 53; u) I. Gunkel, T. Thurn-Albrecht, *Macromolecules* **2012**, *45*, 283; v) C. B. Roth, J. M. Torkelson, *Macromolecules* **2007**, *40*, 3328; w) Y. Matsushita, A. Noro, M. Iinuma, J. Suzuki, H. Ohtani, A. Takano, *Macromolecules* **2003**, *36*, 8074; x) J. Heier, E. J. Kramer, J. Groenewold, G. H. Fredrickson, *Macromolecules* **2000**, *33*, 6060; y) Y.-T. Chen, C.-T. Lo, *Soft Matter* **2013**, *9*, 1756; z) H. Lu, B. Akgun, X. Wei, L. Li, S. K. Satija, T. P. Russell, *Langmuir* **2011**, *27*, 12443.

[96] a) L. S. Grundy, V. E. Lee, N. Li, C. Sosa, W. D. Mulhearn, R. Liu, R. A. Register, A. Nikoubashman, R. K. Prud'homme, A. Z. Panagiotopoulos, R. D. Priestley, *ACS Nano* **2018**, *12*, 4660; b) T. Higuchi, A. Tajima, K. Motoyoshi, H. Yabu, M. Shimomura, *Angew. Chem. Int. Ed.* **2009**, *48*, 5125; c) C. Shin, H. Ahn, E. Kim, D. Y. Ryu, J. Huh, K.-W. Kim, T. P. Russell, *Macromolecules* **2008**, *41*, 9140; d) K. Mita, H. Tanaka, K. Saijo, M. Takenaka, T. Hashimoto, *Polymer* **2008**, *49*, 5146; e) T. Higuchi, A. Tajima, H. Yabu, M. Shimomura, *Soft Matter* **2008**, *4*, 1302; f) T. Higuchi, A. Tajima, K. Motoyoshi, H. Yabu, M. Shimomura, *Angew. Chem. Int. Ed.* **2008**, *47*, 8044; g) J. Yoon, W. Lee, E. L. Thomas, *Adv. Mater.* **2006**, *18*, 2691; h) V. Kalra, S. Mendez, J. H. Lee, H. Nguyen, M. Marquez, Y. L. Joo, *Adv. Mater.* **2006**, *18*, 3299; i) H. Yabu, T. Higuchi, M. Shimomura, *Adv. Mater.* **2005**, *17*, 2062; j) M. J. Fasolka, L. S. Goldner, J. Hwang, A. M. Urbas, P. DeRege, T. Swager, E. L. Thomas, *Phys. Rev. Lett.* **2003**, *90*, 016107; k) F. Court, T. Hashimoto, *Macromolecules* **2001**, *34*, 2536; l) J. H. Laurer, S. D. Smith, J. Samseth, K. Mortensen, R. J. Spontak, *Macromolecules* **1998**, *31*, 4975; m) L. Kane, M. M. Satkowski, S. D. Smith, R. J. Spontak, *Macromolecules* **1996**, *29*, 8862; n) E. Helfand, Z. R. Wasserman, *Macromolecules* **1976**, *9*, 879.

[97] W. Zhang, M. Huang, S. a. Abdullatif, M. Chen, Y. Shao-Horn, J. A. Johnson, *Macromolecules* **2018**, *51*, 6757.
